# Microsurgical skill decay in novices one year after training: a longitudinal follow-up study

**DOI:** 10.1016/j.bas.2026.107723

**Published:** 2026-11-01

**Authors:** Andrei Schildt, Anni Pohjola, Ville Vasankari, Jenni Määttä, Anna Seidlová, Martin Májovský, Norbert Svoboda, David Netuka, Martin Lehecka

**Affiliations:** a Department of Neurosurgery, Helsinki University Hospital and University of Helsinki, P.O. Box 266, FI-00029, Helsinki, Finland; b Department of Neurosurgery and Neurooncology, First Faculty of Medicine, Charles University and Military University Hospital, Prague, Czech Republic

**Keywords:** Skill decay, Neurosurgery, Surgical training, Exoscope

## Abstract

**Introduction:**

Interruptions to clinical practice are common. Skill decay is known to occur during breaks in various surgical specialties. We aim to identify the extent of skill decay following a one-year break after completion of a dedicated microsurgical training course.

**Research question:**

How significantly do microsurgical skills decay one year after training?

**Materials and methods:**

We recruited 25 medical students who completed an 8-h microsurgical training course one year earlier. Microsurgical skills were measured using a standardized test task (12 simple interrupted 8-0 sutures on a 6 mm silicone tube). Each participant completed the task prior to the microsurgical course, post-training, one-year after the course and an additional re-attempt. Performances were analysed based on speed, error counts, accuracy and a qualitative rating.

**Results:**

Comparing the one-year test with the post-training test, we found that significant decay had occurred. Median task time increased from 28.5 to 34.1 min (p < 0.001), total errors increased from 42 to 56 (p < 0.001), and the qualitative score fell from 9 to 7 (p < 0.001). At the immediate re-test, speed recovered to post-training level.

**Discussion and conclusion:**

We found that a one-year interruption in microsurgical training resulted in microsurgical skill decay, reflected by decreased speed and increased error counts. Upon an immediate re-test, speed returned to the post-training level, whereas quality and error counts did not fully recover. These findings indicate that even after long interruptions, microsurgical speed can be rapidly regained to levels comparable to those achieved before the break.

## Highlights

• Surgical skill decay can occur following interruptions to practice.
• We assessed skill decay one year after an 8-h microsurgical training course.
• Skill decay was observed, with increased task completion times and error rates.
• Speed recovered on immediate re-testing; quality and errors did not fully recover.

## 1 Introduction

Interruptions to surgical practice are common. These breaks are usually due to professional-, personal- and system-related reasons (Robertson et al., 2009). Because neurosurgeons heavily depend on their manual skills, the question of how fast these skills decline is of practical concern. This question carries particular weight in microsurgery, where skill maintenance requires either frequent operations or access to a dedicated laboratory or simulation environment.

Skill decay has been previously studied in surgery mainly for laparoscopic skills (Bonrath et al., 2012; Bekele et al., 2019; Rosenthal et al., 2010; de Andres Crespo et al., 2025; Der et al., 2021; Guseila et al., 2014; Jenison et al., 2012; Joosten et al., 2022; Kahol et al., 2010), but only rarely in microsurgery (Grober et al., 2004). Measurable deterioration has been described as early as around eleven weeks to four months (Bonrath et al., 2012; Kahol et al., 2010; Varley et al., 2015), with further decline reported at six months (Kahol et al., 2010; Castellvi et al., 2009; Howells et al., 2009). Although the reported timelines differ, interruptions to training and clinical practice seem to consistently result in some degree of skill decay. Since microsurgery poses its own distinct challenges and demands, findings from other specialties may not translate directly. Establishing a timeline for skill decay in microsurgery is also essential to design evidence-based microsurgical training programs and residency curricula.

The primary aim of this study is to measure the retention of basic microsurgical dexterity in surgical novices, one year after they completed a basic microsurgical course. We hypothesize that a measurable decline will be evident at one year. However, we expect that skills will be rapidly recovered once participants are reintroduced to the task.

## 2 Methods

### 2.1 Study population

For this study, we recruited participants of our previous prospective, multicentre laboratory study on the effectiveness of video-based training for basic microsurgical skill development, which was carried out about one year earlier (Schildt et al., 2026). Participants in the original study were 30 medical students from the University of Helsinki (n = 20) and First Faculty of Medicine at Charles University in Prague (n = 10). All were microsurgical novices without prior surgical experience. They participated in a training program which consisted of weekly 2-h training sessions, lasting 4 weeks, and resulting in a total of 8 h of training. In that study, participants were randomized to receive either hands-on training from experienced surgeons or independent training using video-material only and they used either a surgical microscope (50%) or an exoscope (50%) throughout the course. The results showed that video-based learning resulted in similar learning outcomes as on-site tutoring and magnification device did not affect the learning experience.

All participants of the original training program (n = 30) were sent an invitation to participate in a follow-up study one year later. As part of the follow-up invitation questionnaire, participants were asked to report whether they had gained any microsurgical experience since completing the training course. Participants with more than 1 h of microsuturing experience during that follow-up year were to be excluded from the follow-up study. However, no participants were excluded on this basis, as none met this criterion. All participants gave their informed consent. We adhere to the Declaration of Helsinki.

Of the initial 30 course participants, 25 (83%) returned for the follow-up study. All participants from the University of Helsinki were retained, whereas five participants from Charles University in Prague did not participate in the follow-up study. All of the five non-returners received video-education only in the original course, four had trained using the exoscope and one using the microscope (Fig. 1). Returners and non-returners did not differ significantly at baseline (T0) or immediately post-training (T1) in task time, error count, or qualitative score (all p ≥ 0.13; Supplemental Table 1).

**Fig. 1:**
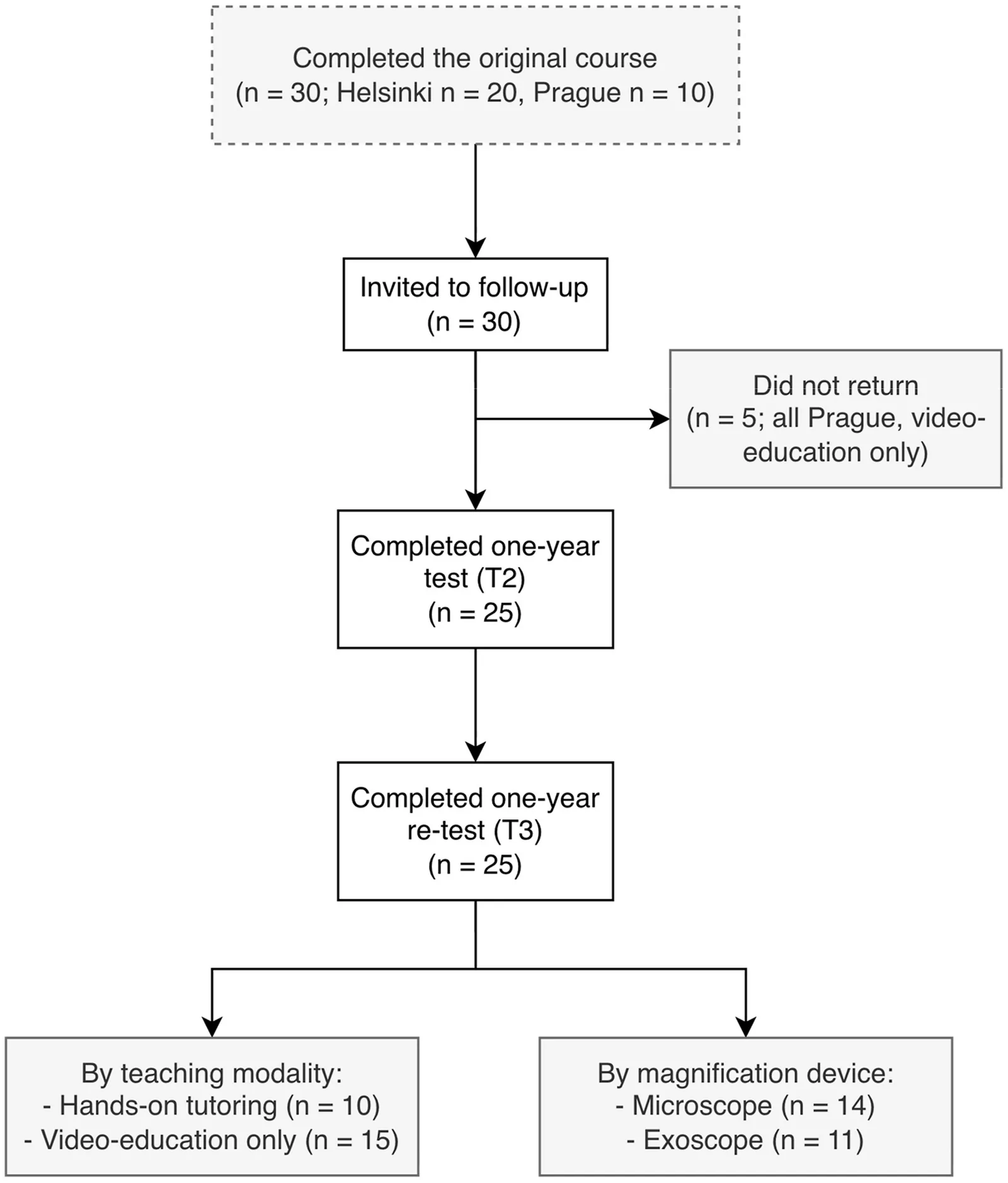
Flowchart of participant selection and attrition throughout the study.

### 2.2 Study protocol

Participants carried out the same microsuturing test task as they did one year before. Prior to the test task, they were given the opportunity to review video teaching material demonstrating microsuturing, and they were reminded of the grading criteria for the task. The instructional videos are in the original publication (Schildt et al., 2026). The test task was completed using the same magnification device (exoscope or microscope) as previously.

After completing the initial follow-up test task (T2), the participants were given the opportunity for a short break of approximately 5 min, after which they carried out the same test task for the second time (T3). The idea was to investigate how much microsurgical dexterity was regained upon a second consecutive trial. In total, each participant completed four test tasks: one prior to participation in the original course (T0), post-training (T1), immediately after the one-year break (T2) and an additional re-attempt (T3).

In the original study, both trials (T0 and T1) were conducted on days separate from the course training sessions. These tests were preceded by a warm-up consisting of six simple interrupted sutures, whereas no warm-up was permitted at the one-year follow-up (T2).

### 2.3 Test task

The test task was identical to the one used to evaluate progression throughout the initial training programme (Schildt et al., 2026). It consisted of suturing a longitudinal slit (slit length 12 mm) on a silicone tube (diameter 6 mm) using 12 simple interrupted 8-0 sutures. For completion of the task, two identical jeweler's forceps and microscissors were used. All test tasks were recorded for further analysis. The scoring parameters were: (i) task completion time, (ii) mean absolute deviation of bite sizes and suture spacings (iii) error counts (e.g. dropping the needle, losing the thread, unnecessarily puncturing the tube), and (iv) a qualitative rating**.** The qualitative rating encompassed four categories: smoothness, respect for tissue, instrument handling and knot tying. Each category was rated from 1 to 3 (total 4-12). All scoring parameters were identical to those in the original study (Schildt et al., 2026). The videos were scored by author A.S., who was blinded to the timepoint and teaching group.

Four timepoints were compared: baseline (T0), post-training (T1), one-year retention (T2), and an immediate re-attempt (T3). Task time was the primary endpoint and error count the key secondary variable; mean absolute deviation (MAD) of bite size, suture spacing, and the total qualitative score were also analysed.

### 2.4 Statistical analysis

Data are presented as median [IQR]. As several variable distributions deviated from normality (Shapiro–Wilk), rank-based tests were used. Decay from T1 to T2 was tested with a one-sided Wilcoxon signed-rank test reflecting the pre-specified hypothesis of deterioration, with p-values Holm-adjusted across the five outcomes. Recovery (T2 to T3) was tested one-sided and return to post-training level (T3 vs. T1) two-sided. Decay by teaching modality and by magnification device was compared using Kruskal–Wallis tests. As an exploratory analysis, one-year performance (T2) was modelled by linear regression with post-training performance (T1) and teaching group as covariates, with adjustment for center (Prague or Helsinki). Attrition was assessed by Mann–Whitney U tests comparing returners and non-returners. The proportion of training gain retained at one year was estimated as 100 × (1 − median decay/median gain), with a 95% bootstrap confidence interval (5000 resamples).

P-values below 0.05 were considered significant. Analyses were performed in R (version 4.4.0) using RStudio (version 2024.12.0.467).

## 3 Results

### 3.1 Skill decay after one year

Comparing the one-year test (T2) with the post-training test (T1), we found that significant decay had occurred. Task time increased by a median of 6 min (p < 0.001), total errors rose by a median of 13 errors (p < 0.001), and the qualitative score fell by a median of two points (p < 0.001). All three effects survived Holm correction across the five outcomes and remained significant under two-sided testing (all p < 0.005). By category, the increase in errors consisted primarily of needle drops (median 2 to 10 errors, p < 0.001) and thread drops (14 to 22, p = 0.002). Unnecessary penetrations of the tube did not increase (9 vs. 10, p = 0.506; Supplemental Table 2).

In contrast, neither quality metric derived from mean absolute deviation showed significant decay (MAD bite size p = 0.72; MAD suture spacing p = 0.11); these metrics had not shown significant training gain between T0 and T1 (p = 0.075 and 0.059), so their retention could not be meaningfully quantified. Despite reaching statistical significance, the decay was modest in magnitude relative to the initial training gain: participants retained an estimated 76% (95% CI 60–86%) of their speed improvement, 68% (25–92%) of their error reduction, and 60% (25–75%) of their qualitative rating gain from T0 to T1. Performance at each of the four timepoints (T0-T3) is visualized in Fig. 2 and full comparisons of decay between T1 and T2 are summarised in Table 1.

**Fig. 2:**
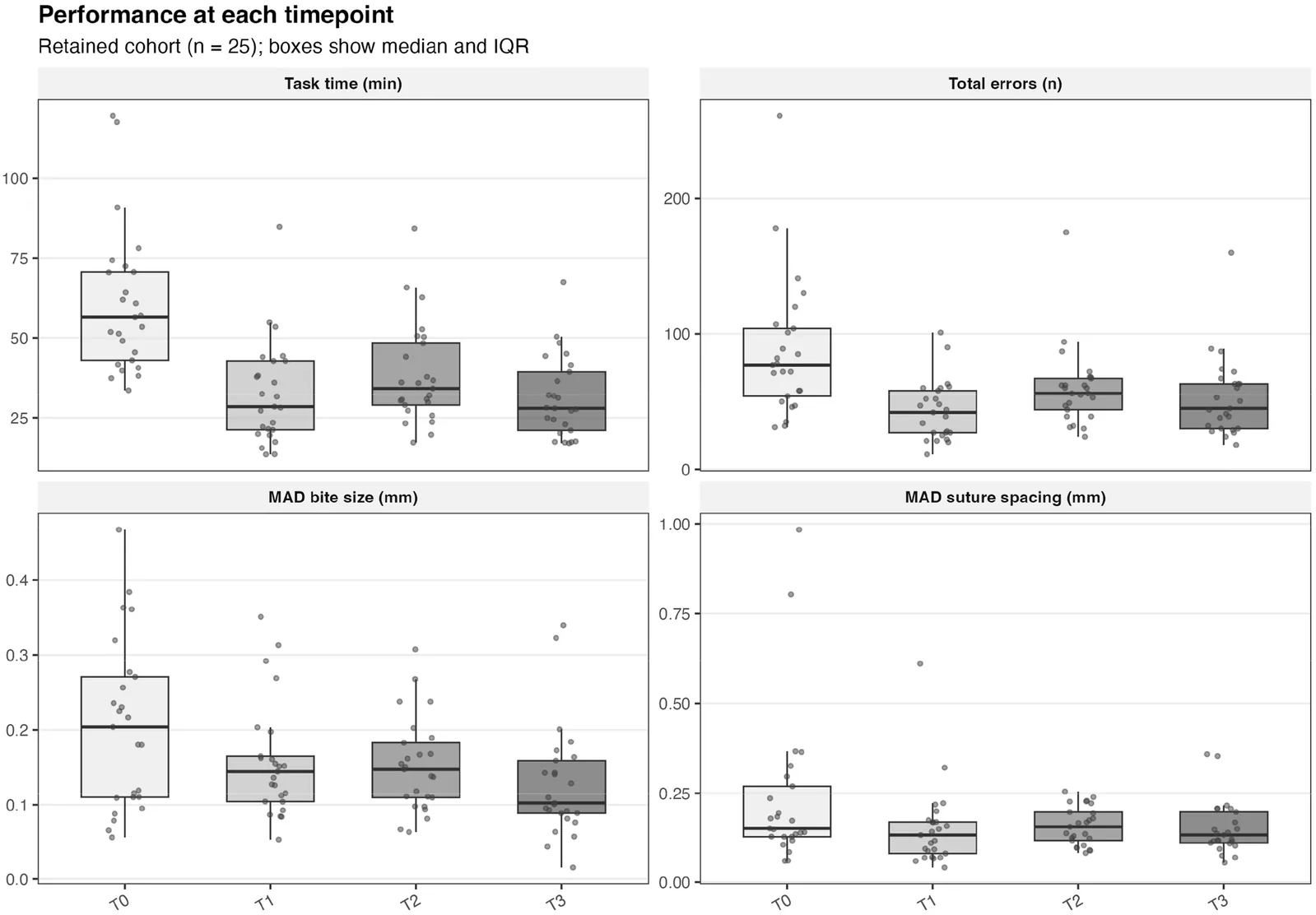
Performance across each of the timepoints. T0 = baseline, T1 = post-training, T2 = one year after training, T3 = re-attempt.

**Table 1:** Within-subject training gain, one-year decay and retained fraction (retained cohort, n = 25). Median [IQR] of within-participant differences are positive in the direction named in each column heading (improvement for gain, deterioration for decay).

Outcome	Training gain (T0 to T1)	P-value	Decay (T1 to T2)	P-value	% gain from T0 to T1 retained at T2 [95% CI]
Task time (min)	25.6 [20.0–30.1]	**<0.001**	6.1 [3.6–9.8]	**<0.001**	76 [60, 86]
Total errors (n)	41 [14–56]	**<0.001**	13 [4–28]	**<0.001**	68 [25, 92]
MAD bite size (mm)	0.038 [-0.035–0.125]	0.075	−0.020 [-0.047–0.036]	0.720	N/A
MAD suture spacing (mm)	0.036 [-0.023–0.147]	0.059	0.021 [-0.025–0.069]	0.110	N/A
Qualitative score (total)	5.0 [3.0–5.0]	**<0.001**	2.0 [1.0–3.0]	**<0.001**	60 [25, 75]

### 3.2 Recovery after re-exposure

A single immediate re-attempt (T2 to T3) largely reversed the one-year skill decay in suturing speed. Task time improved by a median of 6 min on re-attempt (one-sided p < 0.001) with no significant difference between re-attempt and original post-training performance (T3 vs. T1, p = 0.63). The qualitative score also improved on re-attempt (p = 0.003) but remained significantly below the post-training level (p = 0.002). Error counts also recovered significantly (recovery p = 0.035; re-attempt vs. post-training p = 0.17) but remained a median of five errors above post-training. Results are shown in Table 2.

**Table 2:** Recovery on the immediate re-attempt (T2 to T3) and comparison with post-training performance (T3 vs. T1). Median [IQR] of within-participant differences are positive in the direction named in each column heading (improvement for recovery, worse than T1 for deficit).

Outcome	Recovery (T2 to T3)	P-value	Residual deficit at T3 (vs. T1)	P-value
Task time (min)	5.8 [2.9–9.8]	**<0.001**	−0.6 [-4.4–3.5]	0.634
Total errors (n)	2 [-2–18]	**0.035**	5 [-6–14]	0.170
MAD bite size (mm)	0.015 [-0.021–0.044]	0.082	−0.029 [-0.074–0.008]	0.085
MAD suture spacing (mm)	0.014 [-0.033–0.048]	0.205	0.021 [-0.035–0.054]	0.458
Qualitative score (total)	1.0 [0.0–2.0]	**0.003**	1.0 [0.0-3.0]	**0.002**

### 3.3 Effect of teaching modality and magnification device

We attempted to examine the effect of video-education and magnification device (exoscope or microscope) on skill decay. However, due to the small number of participants and all dropouts being from the video-education only group, the analyses should be regarded as exploratory only. When comparing the participants of the follow-up study at T1, the subgroups did not differ significantly in task time (tutor 28.3 vs. video 32.5 min, p = 0.781), errors (50 vs. 28 errors, p = 0.120) or qualitative score (8.5 vs. 9.0, p = 0.571), although the tutor group had more errors. At one year, however, participants who originally trained with video-education only showed greater one-year decay (T2 vs. T1) than those who had received hands-on tutoring in task time (median decay 9.1 vs. 3.9 min; p = 0.009) and in error count (18 vs. 4; p = 0.020). The qualitative score did not differ (p = 0.51). In contrast, the magnification device (exoscope vs. microscope) was not associated with decay in any outcome (all p ≥ 0.22; Table 3).

**Table 3:** One-year decay (T1 to T2) by teaching modality and magnification device (retained cohort, n = 25). Median [IQR] of within-participant decay from T1 to T2, positive values denote deterioration.

Outcome	Tutor decay (n = 10)	Video decay (n = 15)	P-value (video vs. tutor)	Exoscope decay (n = 11)	Microscope decay (n = 14)	P-value (exoscope vs. microscope)
Task time (min)	3.9 [2.9–5.5]	9.1 [5.8–10.2]	**0.009**	6.9 [3.9–9.6]	5.2 [2.9–9.6]	0.511
Total errors (n)	4 [-6–12]	18 [8–38]	**0.020**	8 [3–16]	15.5 [4.3–37.0]	0.366
Qualitative score (total)	2.0 [1.0–3.0]	2.0 [1.5–3.5]	0.514	3.0 [1.5–3.5]	2.0 [1.0–2.8]	0.229

In a linear regression of T2 performance on T1 performance and teaching group, with adjustment for the center (Prague or Helsinki), the video group was slower than the tutor group at one year (5.5 min, 95% CI 1.0 to 10.0, p = 0.020), whereas the difference in errors was no longer significant (p = 0.372).

Comparing the immediate re-test with post-training performance (T3 vs. T1), the difference between modality groups (video vs. hands-on) was no longer significant for speed (1.0 vs. −3.1 min; p = 0.052), whereas differences persisted for error count (11 vs. −4; p = 0.016) and qualitative score (2.0 vs. 0.0; p = 0.028).

## 4 Discussion

In line with our hypotheses, we observed decay in microsurgical dexterity following a one-year break from training, particularly in task completion speed, qualitative rating and the number of errors committed. Upon an immediate re-test, most of the observed decline in speed was reversed, whereas other metrics remained below the post-training level. These findings show that significant skill decay does occur, but that performing a second consecutive trial of the same task partially mitigates the decline.

Previous studies evaluating skill decay in non-microsurgical specialties have reported partly conflicting findings, primarily in basic surgical skills and laparoscopic simulators. Bekele et al. reported significant decay of simple interrupted suturing and knot-tying at both six months and one year in 44 medical students (Bekele et al., 2019), whereas proficiency-based laparoscopic training has been reported to produce durable performance retention at up to one year (Rosenthal et al., 2010). The majority of studies nonetheless indicate that some measurable decay follows breaks longer than roughly three months (Bonrath et al., 2012; Der et al., 2021; Guseila et al., 2014; Jenison et al., 2012; Joosten et al., 2022; Kahol et al., 2010). Our findings largely align with this literature, expanding it to microsurgery: participants retained an estimated 76% of the speed improvement gained during the training course (T0 to T1), 68% of error reduction and 60% of qualitative score gain at the one-year task (T2). On an immediate re-attempt, speed recovered rapidly, while the qualitative score and error count did not fully recover.

Studies of microsurgical training have mainly described the learning curve during training (Lefevre et al., 2022). One study followed 44 trainees through a seven-month microsurgical anastomosis training program to establish the learning curve for microsurgical skills (Lefevre et al., 2022). The authors found that the anastomosis patency rate peaked at 70%, thereafter reaching a plateau. In contrast to our study, however, only the initial phase of training was considered and no longitudinal data on skill retention were collected. Our findings show that this later phase deserves equal attention, and that microsurgical training programmes could include periodic re-certification, for example a standardized bench test at fixed intervals with refresher training when performance falls below a predefined standard.

The improvement in speed, error counts and qualitative ratings likely reflects a warm-up effect elicited by the first re-test (T2). Previous studies have repeatedly shown that short warm-up practice prior to surgery leads to improved performance, particularly for laparoscopic and robotic surgery (Abdalla et al., 2015; Calatayud et al., 2010; Lendvay et al., 2013; Chen et al., 2013; de Araujo Neto et al., 2026). Our findings suggest that a similar phenomenon may exist in microsurgery and that incorporating a warm-up may be sufficient to compensate for much of the skill decay that develops after a prolonged interruption. On the other hand, some of the observed skill decay may be attributable to the absence of a warm-up, meaning that T3 may more accurately reflect the extent of decay after a one-year break. In practice, this suggests that the magnitude of skill decay may be smaller than initially observed.

Our exploratory analyses raise the hypothesis that tutor-based learning may lead to better long-term skill retention. But, due to the relatively small participant counts, the result should be treated with caution and requires further investigation, in the form of a larger, adequately powered study. Earlier studies indicate that tutor-based teaching of microsurgical skills produces similar results to training using purely video-based independent practice (Schildt et al., 2026; Chien et al., 2015; Nousiainen et al., 2008; Dąbrowski et al., 2022). In our follow-up cohort, however, participants who had received video-based training experienced significantly more decay in speed at the one-year follow-up (T2). It is feasible that this effect could be due to better recollection of personalized training compared to independent practice using only videos.

Our study has limitations. First, participants likely differ in terms of the manual practice they have received through internships and courses. However, no participants reported additional microsuturing experience exceeding 1 hour following the course. Nevertheless, as macrosurgical skills share some similarities with microsuturing, other surgical rotations may have confounded the results by attenuating the observed skill decay. Second, our relatively small sample size may limit the generalisability of our findings. Third, it should also be noted that all of the participants who dropped out were from the cohort recruited from Charles University in Prague. While this factor is unlikely to have influenced our main analysis assessing skill decay, it may have impacted our subgroup analyses comparing teaching modality and magnification device. As such, our subgroup analyses should be regarded as exploratory. Fourth, the cohort consisted entirely of medical students, for whom initial gains are large and as such skill decay is likely also greater, meaning that changes are more readily detectable compared to experienced surgeons. In general, the dynamics of skill decay in novices may not entirely transfer to seasoned practitioners. Fifth, the rapid regains in skill could be due to the task being relatively easy. It is possible that proficiency in more difficult tasks would not be regained. Sixth, all recordings were assessed by a single rater, and as such inter-rater agreement could not be established. Also, due to differences in the formatting of exoscopic and microscopic videos, blinding to the magnification device was not possible. Lastly, as the MADs of bite size and suture spacing showed neither improvement after training nor deterioration, these metrics may not be sufficiently sensitive to capture changes in microsurgical skills.

## 5 Conclusion

We found that a one-year interruption in microsurgical training resulted in microsurgical skill decay, reflected by decreased speed, qualitative rating and increased error counts. Upon an immediate re-test of the same task, speed returned to the post-training level, whereas quality and error counts did not fully recover. These findings indicate that even after long interruptions, microsurgical speed can be rapidly regained to levels comparable to those achieved before the break, while other aspects (quality, errors) may require longer retraining.

## Ethics declaration

Written informed consent to take part in the study and to publish the article has been obtained from all participants or their legal representatives. The privacy rights of participants have been observed.

This study was performed in compliance with relevant laws, regulatory frameworks and guidelines where the research took place. Ethics committee approval was not required under relevant laws and institutional guidelines. The study involved healthy adult volunteers performing a microsuturing task on a synthetic model, with no patients, patient data, biological samples or intervention in participants' physical or psychological integrity. It was conducted in accordance with the Declaration of Helsinki.

## Declaration of competing interest

The authors declare that they have no known competing financial interests or personal relationships that could have appeared to influence the work reported in this paper.
