# Supplementary material for: Microsurgical skill decay in novices one year after training: a longitudinal follow-up study
Source: Brain Spine. 2026 Nov 1;6:107723. doi: 10.1016/j.bas.2026.107723 (PMC13756518; doi:10.1016/j.bas.2026.107723)
Supplement: Supplementary file 1 [file mmc1.docx]

Supplemental Table 1. Baseline and post-training differences between those participants who returned for the one-year follow-up and those who did not.

| **Outcome** | **Timepoint** | **Returners**  **(n=25)** | **Non-returners**  **(n=5)** | **P-value** |
| --- | --- | --- | --- | --- |
| Task time (min) | T0 | 56.5 [43.0–70.6] | 56.4 [40.2–61.2] | 0.914 |
|  | T1 | 28.5 [21.2–42.8] | 37.7 [31.1–61.0] | 0.133 |
| Total errors (n) | T0 | 77 [54–104] | 75 [58–77] | 0.802 |
|  | T1 | 42 [27–58] | 45 [27–49] | 0.956 |
| Qualitative score (total) | T0 | 4.0 [4.0–5.0] | 4.0 [4.0–4.0] | 0.442 |
|  | T1 | 9.0 [8.0–10.0] | 8.0 [8.0–10.0] | 1.000 |
