# Supplementary material for: Microsurgical skill decay in novices one year after training: a longitudinal follow-up study
Source: Brain Spine. 2026 Nov 1;6:107723. doi: 10.1016/j.bas.2026.107723 (PMC13756518; doi:10.1016/j.bas.2026.107723)
Supplement: Supplementary file 2 [file mmc2.docx]

**Supplemental Table 2.** Errors by category at each time point (retained cohort, n = 25). Values are median [IQR].

| **Error category** | **T0** | **T1** | **T2** | **T3** | **P (T0 to T1)** | **P (T1 to T2)** | **P (T2 to T3)** | **P (T3 vs. T1)** |
| --- | --- | --- | --- | --- | --- | --- | --- | --- |
| **Unnecessary penetration of tube** | 11 [8–17] | 9 [5–15] | 10 [5–17] | 7 [4–12] | 0.567 | 0.506 | 0.154 | 0.229 |
| **Needle drops** | 7 [3–11] | 2 [1–5] | 10 [4–15] | 6 [2–13] | 0.011 | <0.001 | 0.078 | 0.034 |
| **Losing the thread** | 38 [28–55] | 14 [9–20] | 22 [12–28] | 19 [14–28] | <0.001 | 0.002 | 0.121 | 0.150 |
| **Both instruments out of view** | 10 [5–18] | 11 [5–14] | 14 [9–17] | 11 [7–17] | 0.657 | 0.070 | 0.161 | 0.925 |
| **Total errors** | 77 [54–104] | 42 [27–58] | 56 [44–67] | 45 [30–63] | <0.001 | <0.001 | 0.035 | 0.170 |
